# Extra energy for hearts with a genetic defect: ENERGY trial

**DOI:** 10.1007/s12471-019-1239-0

**Published:** 2019-02-14

**Authors:** B. O. van Driel, A. C. van Rossum, M. Michels, R. Huurman, J. van der Velden

**Affiliations:** 1 Department of Physiology, Amsterdam Cardiovascular Sciences, Amsterdam UMC, location VUmc, Amsterdam, The Netherlands; 2 Department of Cardiology, Amsterdam Cardiovascular Sciences, Amsterdam UMC, location VUmc, Amsterdam, The Netherlands; 3 Department of Cardiology, Erasmus Medical Center Rotterdam, Rotterdam, The Netherlands

**Keywords:** Hypertrophic cardiomyopathy, Metabolic preventive therapy, Trimetazidine

## Abstract

**Aims:**

Previous studies have shown that hypertrophic cardiomyopathy mutation carriers have a decreased myocardial energy efficiency, which is thought to play a key role in the pathomechanism of hypertrophic cardiomyopathy (HCM). The ENERGY trial aims to determine whether metabolic drugs correct decreased myocardial energy efficiency in HCM mutation carriers at an early disease stage.

**Methods:**

40 genotype-positive, phenotype-negative *MYH7* mutation carriers will be treated for two months with trimetazidine or placebo in a double-blind randomised study design. Directly before and after treatment, study subjects will undergo an [^11^C]-acetate positron emission tomography/computed tomography (PET/CT) and cardiac magnetic resonance (CMR) scan to measure myocardial energy efficiency. Myocardial efficiency will be calculated as the amount of oxygen the heart consumes to perform work.

**Conclusion:**

The ENERGY trial will be the first proof of concept study to determine whether metabolic drugs are a potential preventive therapy for HCM. Given that trimetazidine is already being used in clinical practice, there is large potential to swiftly implement this drug in HCM therapy.

## Introduction

### Background on hypertrophic cardiomyopathy

Hypertrophic cardiomyopathy (HCM) is the most common genetic cardiomyopathy with an estimated prevalence of 1:500 to 1:200 [1–4]. It is a cause of acute cardiac arrest at a young age and cardiac transplantation in end-stage heart failure patients [1, 2]. As familial HCM is an autosomal dominant genetic disorder, most patients are heterozygous for the mutation and carry one mutant and one normal allele. Even though the gene mutation is present at birth, disease onset generally occurs between 20 and 50 years of age, thus affecting patients in the prime of their life. The sarcomeric gene mutation causing HCM was first identified more than 25 years ago. Currently >1,400 HCM mutations have been found, of which ~90% reside in the genes encoding thick and thin filament proteins of the sarcomere [4]. The 3 most frequently affected genes (*MYH7, MYBPC3* and *TNNT2*) encode the sarcomeric proteins myosin heavy chain (MyHC), myosin-binding protein C (cMyBP-C) and troponin T (cTnT) [2, 4]. With the advance in genetic screening, more genotype-positive phenotype-negative (G+/Ph-) individuals are currently being identified, which increases the number of people that are insecure of their fate as treatment to prevent disease is lacking [5].

### Inefficient cardiac pump function as target for therapy: proof from preclinical and clinical studies

**Fig. 1:**
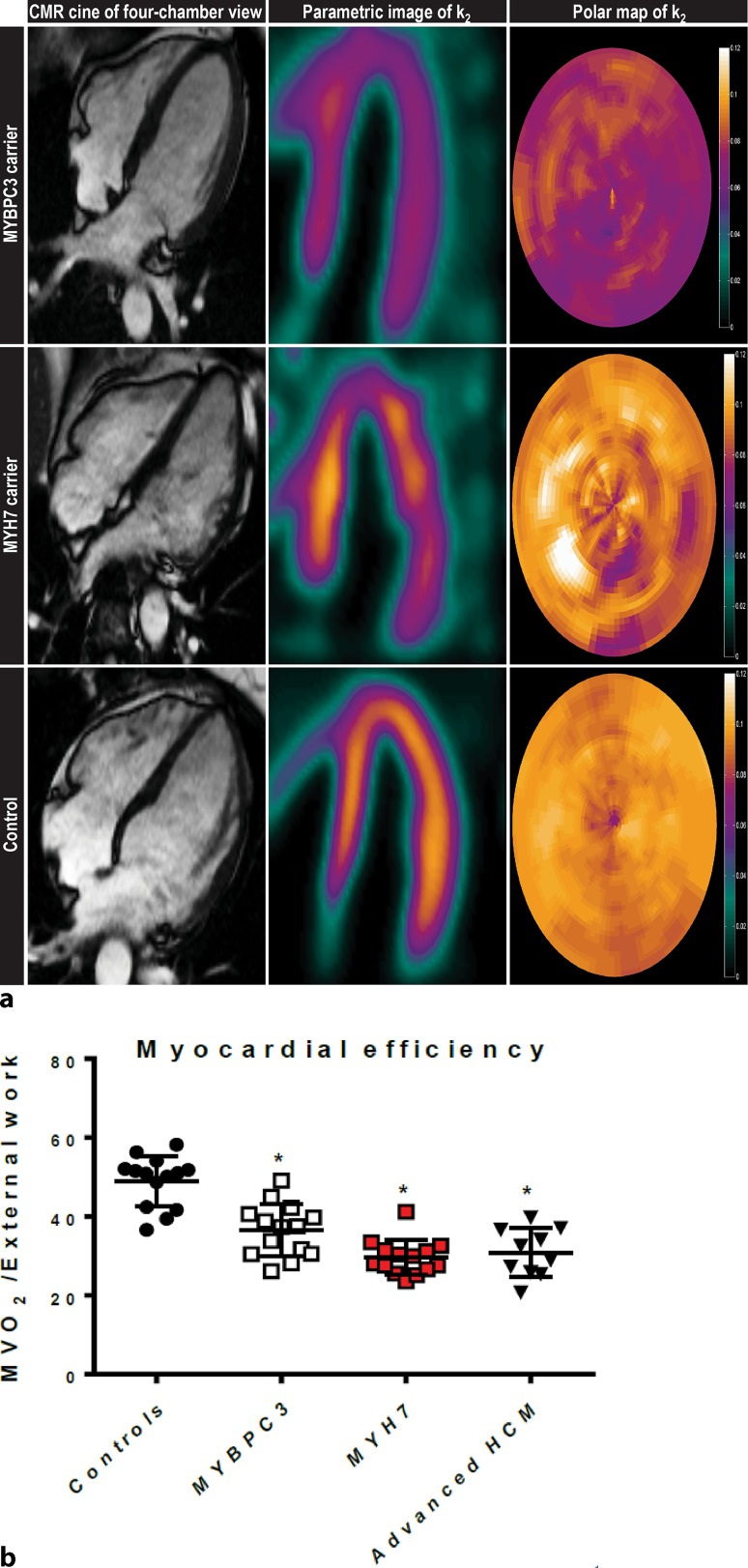
The ratio between external work and myocardial oxygen consumption to obtain myocardial external efficiency was determined in 28 asymptomatic mutation carriers (14 *MYBPC3* and 14 *MYH7*), 10 manifest HCM patients and 14 healthy controls using [^11^C]-acetate positron emission tomography (PET) and cardiovascular magnetic resonance imaging (CMR). **a** CMR-derived cardiac 4‑chamber view and parametric images of [^11^C]-acetate PET derived k2 with corresponding polar maps. As can be seen clearly, oxygen metabolism was higher in asymptomatic mutation carriers compared to controls [7]. **b** Myocardial efficiency is not further reduced in advanced HCM patients compared with mutation carriers [8]

Using metabolic therapy we aim to reverse deficits in cardiac energetics, which are observed at the very early disease stage in seemingly healthy (asymptomatic) mutation carriers (Fig. 1). A reduced ratio between phosphocreatine and adenosine triphosphate (PCr/ATP), a measure of energetic status of the heart, was observed in mutation carriers without hypertrophy [6]. In addition, myocardial external efficiency was significantly reduced in asymptomatic individuals with mutations in genes encoding thick filament proteins [7]. Imaging studies in HCM patients with advanced disease showed that myocardial efficiency did not further decline compared to asymptomatic mutation carriers, indicating that energy deficiency is an early change in the heart before the development of hypertrophy [8]. Research data has suggested that at cardiac muscle cell level, two mutation-mediated changes in myofilament function underlie reduced cardiac efficiency in HCM. Firstly, the mutation-induced increase in ATP utilisation of sarcomeres, which was reported in transgenic HCM mice [9]. In line with these findings, our studies in cardiac tissue from HCM patients showed an increased cost of muscle contraction, illustrated by a ~ 2-fold higher tension cost (ratio between ATP utilisation and generated force) compared with controls [7, 10]. Secondly, a mutation-related increase in myofilament calcium sensitivity was found in HCM mouse models [11], which increases both force development and ATP consumption. In accordance with HCM mouse models, studies in human cardiac tissue revealed higher calcium sensitivity in HCM human cardiac tissue compared with controls [12, 13]. Overall, these studies show that inefficient cardiac pump function and altered energetic status occur at an early disease stage even before development of cardiac hypertrophy, and may thus represent a target for preventive therapy.

### Metabolic therapy as potential preventive therapy in HCM

Metabolic agents, which are already used in clinical practice such as anti-anginal agents, may be used to improve cardiac energetics and function [14–19]. In the healthy heart energy demand is met by oxidation of fatty acids and carbohydrates. Although fatty acids represent the predominant fuel for the heart, they provide less ATP per O_2_ molecule in comparison to carbohydrates [14]. Thus, agents which shift metabolism away from the preferred fatty acids towards carbohydrates would increase ATP supply and may prevent cardiac hypertrophy and failure. Abozguia et al. showed that metabolic therapy with perhexiline had a beneficial effect in advanced HCM [20]. We recently demonstrated that myocardial efficiency is already reduced in healthy human mutation carriers [7, 10]. Therefore, optimising the energetic status of the heart may benefit mutation carriers at a very early disease stage before hypertrophy is evident. The ENERGY trial will be a proof of concept study which builds on these previous preclinical and clinical studies. We aim to determine whether metabolic therapy has the potential to restore the reduced cardiac efficiency in HCM mutation carriers.

### Metabolic therapy with trimetazidine

Trimetazidine is an anti-anginal drug, indicated as add-on therapy for the symptomatic treatment of stable angina pectoris in adults who are inadequately controlled by first-line therapies. Trimetazidine inhibits β‑oxidation of fatty acids by blocking the mitochondrial long-chain 3‑ketoacyl coenzyme A thiolase enzyme in the mitochondria [21]. Consequently, glucose oxidation is stimulated which yields more ATP per molecule O2, resulting in a more oxygen-efficient metabolism. The improved energy efficiency obtained with trimetazidine has the potential to correct the energy deficiency observed in HCM. As both *ex vivo* and *in vivo* studies showed a more severe energy deficiency in *MYH7* compared with *MYBPC3* mutations, we will include asymptomatic *MYH7* mutation carriers in the ENERGY trial. This clinical trial will be a proof of concept study to determine whether trimetazidine can improve the myocardial energy efficiency of asymptomatic *MYH7* mutation carriers, as measured by [^11^C]-acetate positron emission tomography/computed tomography (PET/CT) and cardiac magnetic resonance (CMR) scans.

## Study design

**Fig. 2:**
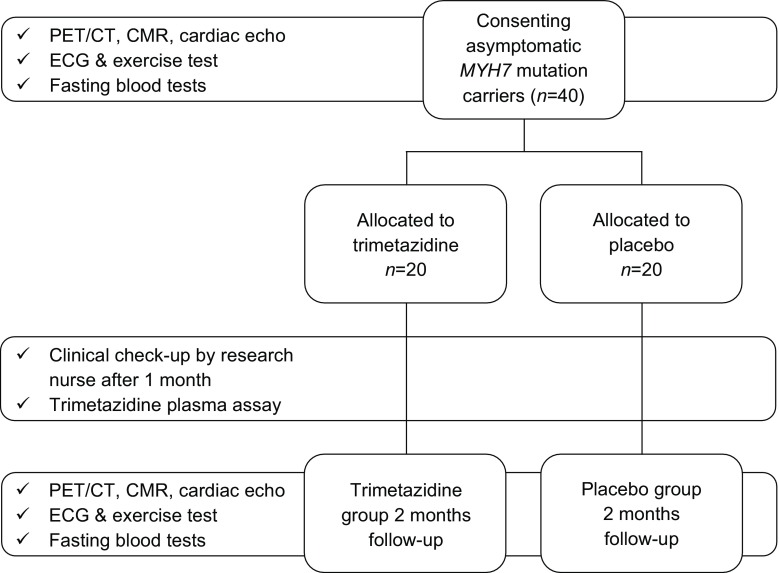
ENERGY trial study design

The ENERGY trial is a randomised, double-blind, placebo-controlled trial. A flow chart of the trial can be found in Fig. 2. Randomisation will be done by randomised block randomisation stratified for the participants’ sex.

### Methods

#### Ethics

This study will be conducted in accordance with the guidelines for Good Clinical Practice. The study will be performed in accordance with the Declaration of Helsinki (64^th^ WMA General Assembly, Fortaleza, Brazil, October 2013). The study has been approved by the Medical Ethical Testing Committee of the VU medical centre.

#### Myocardial efficiency

Myocardial efficiency is the amount of oxygen the heart consumes to perform work. [^11^C]-acetate PET/CT imaging will be used to indirectly quantify myocardial oxygen consumption (MVO_2_). CMR will be performed to calculate cardiac mechanical external work (EW), which is the product of stroke volume and mean arterial pressure. With MVO_2_ and EW, myocardial efficiency can be calculated using the following equation:

$$MEE=\frac{EW\cdot HR\cdot 1.33\times 10^{-4}}{\mathrm{MVO}_{2}\cdot LVM\cdot 20}$$

MEE: myocardial energy efficiency
HR: heart rate
LVM: left ventricular mass

#### Study objectives

##### Primary objective

To measure the effect of trimetazidine on myocardial external efficiency in asymptomatic *MYH7* mutation carriers.

##### Secondary objectives

To study the effect of trimetazidine in asymptomatic *MYH7* mutation carriers on:

- cardiac diastolic function, as measured by echocardiography
- volume parameters of the left ventricle of the heart, measured by CMR.
- exercise capacity: duration of exercise and performed workload
- electrophysiological properties of the heart, measured by ECG

#### Study population

Potential study subjects will be recruited in the HCM outpatient clinic in the Erasmus Medical Center Rotterdam. Asymptomatic carriers of a class 5 MYH7 mutation (definite disease causing mutations) will be asked to participate by their clinician. Recruitment will start in January 2019 until 40 study subjects have been included in the study. After written consent is obtained, participants will be screened for inclusion and exclusion criteria and will be included in the study if they meet the requirements.

##### Inclusion criteria

In order to be eligible to participate in this study, a subject must meet all of the following criteria:

- 18–65 years old
- Class 5* MYH7* mutation identified by pre-symptomatic genetic screening

##### Exclusion criteria

A potential subject who meets any of the following criteria will be excluded from participation in this study:

- Cardiovascular disease
- Hypertrophic cardiomyopathy (maximal wall thickness ≥ 15 mm)
- Wall motion disorders
- Diabetes mellitus
- Any absolute or relative contra-indication for magnetic resonance imaging (MRI) (i. e. metallic implants and claustrophobia)
- Inability to give informed consent.
- Severely impaired renal function with a glomerular filtration rate (GFR) < 30 ml/min
- Parkinson’s disease and related symptoms

#### Sample size calculation

The high sensitivity of the methodology enables to study effects of therapy in a relatively small study group. To reveal a 15% beneficial effect of trimetazidine on myocardial energy efficiency, taking into account a standard deviation of 15% based on our previous studies of myocardial energy efficiency in HCM mutation carriers [7], a number of 20 carriers should be included in each group (with metabolic therapy or placebo) to reach a power of 80% (significance level 0.05).

#### Statistical analysis

Primary and secondary study parameters are all quantitative, continuous variables. Variables will be summarised by means and standard deviation or median and interquartile range, depending on whether they or normally distributed or not. Normality will be assessed using normal probability plots. The primary analysis will be based on intention-to-treat. Missing data will not be imputed. We will use methods, such as mixed model analyses, that are valid under the assumption that data is missing at random. Results will be considered statistically significant with a two-tailed *p*-value < 0.05.

The primary study parameter will be analysed with an ANCOVA analysis. A linear regression model will be used with measurements on day 61 as the dependent variable and treatment group (trimetazidine or placebo group) and the baseline (day 0) measurement as independent variables. The secondary study parameters will be analysed in a mixed model analysis. The models will include treatment group, time point, and interaction as fixed independent variables. The models will also include a random effect for subject.

## Discussion

The ENERGY trial will be the first proof of concept study to determine whether metabolic drugs correct decreased myocardial energy efficiency in asymptomatic mutation carriers at an early disease stage. After giving informed consent, 40 asymptomatic, pre-hypertrophic *MYH7* mutation carriers will be treated for two months with trimetazidine or placebo. Directly before and after treatment, study subjects will undergo an [^11^C]-acetate PET/CT and CMR scan to measure myocardial energy efficiency. A positive result of this trial will help to build proof that metabolic therapy has the potential to become a preventive therapy for HCM. Given that trimetazidine is already used in clinical practice, there is large potential to swiftly implement this drug in HCM therapy.

Trimetazidine is an anti-anginal drug that does not alter the haemodynamic state of the body. Its cardioprotective effect is not yet fully understood, but has been suggested to improve the metabolic state of the heart through a number of mechanisms, such as improvement of energy efficiency by inhibition of free fatty acid β‑oxidation, inhibition of fibrosis, and reduction of oxidative stress, calcium overload and acidosis in the cardiomyocyte [15, 22–24]. Side effects of trimetazidine are mostly mild and well tolerated, with nausea, vomiting and minor headaches being most common. However, in 2011 the European Medicines Agency (EMA) initiated a safety assessment of trimetazidine after a study with trimetazidine reported Parkinson’s disease and related symptoms in 43% of patients [25]. A cumulative review was performed for all spontaneous adverse drug reactions from 1964 to 2011, which estimated the occurrence of this side effect to be 0.36/100,000 patient-years, and reversible in almost 80% of cases [26]. The EMA concluded that the benefits of trimetazidine outweigh the risks in patients with angina pectoris, but that trimetazidine is no longer authorized in the treatment of tinnitus, vertigo or visual field disturbances. Furthermore, use of trimetazidine should be contra-indicated in patients with Parkinson’s disease and other related movement disorders, and in patients with severe renal impairment (GFR < 30 ml/min) [26].

Advancements in genetic screening have led to the identification of a group of individuals, the G+/Ph-group, at risk for developing HCM. This has paved the way for research into therapeutic strategies such as metabolic therapy to prevent the development of HCM in those at risk. New therapeutic strategies emerge as new aspects of HCM pathophysiology are discovered. However, it is unclear whether the different mutations that cause HCM have the same pathomechanism, or that each mutation has its own distinct pathway to disease. This is especially relevant for potential therapeutic strategies, which might only work in patients with a specific mutation. Clinical trials might lead to disappointing results when patients with different HCM mutations are pooled into the same treatment category. For this reason, the ENERGY trial will only include *MYH7* mutation carriers, based on previous research which showed that *MYH7* mutation carriers have the most severe decrease in myocardial energy efficiency [7]. Moreover, it is still unknown why some individuals in the G+/Ph-group never develop HCM, while others develop the disease early in life. If trimetazidine has the potential to become a preventive therapy, development of strategies to accurately identify those at high risk of developing HCM will be crucial to determine who will benefit from preventive therapy.
