# Correction to: Extra energy for hearts with a genetic defect: ENERGY trial

**DOI:** 10.1007/s12471-019-1263-0

**Published:** 2019-03-20

**Authors:** B. O. van Driel, A. C. van Rossum, M. Michels, R. Huurman, J. van der Velden

**Affiliations:** 1 Department of Physiology, Amsterdam Cardiovascular Sciences, Amsterdam UMC, location VUmc, Amsterdam, The Netherlands; 2 Department of Cardiology, Amsterdam UMC, location VUmc, Amsterdam, The Netherlands; 3 Department of Cardiology, Erasmus Medical Center Rotterdam, Rotterdam, The Netherlands

**Correction to:**

**Neth Heart J 2019**

10.1007/s12471-019-1239-0

In the version of the article originally published online, there was an error in Fig. 1a. In the 3 × 3 panel, the images indicated as ‘CMR cine of four-chamber view’, ‘Parametric image of k2’ and ‘Polar map of k2’ were incorrectly matched to the rows indicated as Control, MYBPC3 carrier and MYH7 carrier. The image should have shown the lowest O2 consumption (most purple) images for Control and highest O2 consumption (most orange) for MYH7 carriers. The corrected figure is given below.

**Fig. 1:**
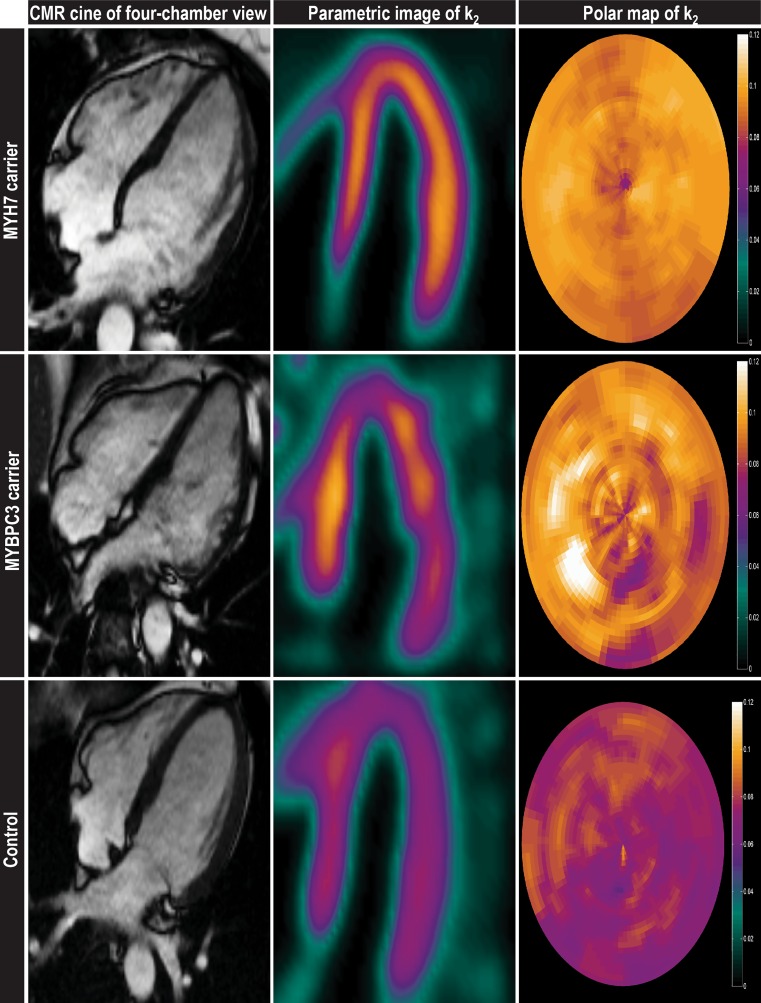
The ratio between external work and myocardial oxygen consumption to obtain myocardial external efficiency was determined in 28 asymptomatic mutation carriers (14 *MYBPC3* and 14 *MYH7*), 10 manifest HCM patients and 14 healthy controls using [^11^C]-acetate positron emission tomography (PET) and cardiovascular magnetic resonance imaging (CMR). **a** CMR-derived cardiac 4‑chamber view and parametric images of [^11^C]-acetate PET derived k2 with corresponding polar maps. As can be seen clearly, oxygen metabolism was higher in asymptomatic mutation carriers compared to controls [7]. **b** Myocardial efficiency is not further reduced in advanced HCM patients compared with mutation carriers [8]

